# Expanding Roles for Muscle Satellite Cells in Exercise-Induced Hypertrophy

**DOI:** 10.1093/function/zqaa040

**Published:** 2020-12-15

**Authors:** Thomas J Hawke

**Affiliations:** Department of Pathology and Molecular Medicine, McMaster University, Hamilton, ON, Canada

## A Perspective on “Satellite Cell Depletion Disrupts Transcriptional Coordination and Muscle Adaptation to Exercise”

The latest work, led by Dr. Davis Englund, from the Charlotte Peterson team at the University of Kentucky in the current issue of *Function*^1^ has made us rethink how we view the role of satellite cells in skeletal muscle adaptation. Since the first microscopic identification by Mauro in 1961^2^ muscle biologists have been fascinated with the role(s), regulation, and therapeutic potential of this muscle stem/progenitor cell population.

Early studies implicated satellite cells as critical contributors to muscle growth, repair, maintenance, and hypertrophy; with many comprehensive reviews highlighting these original works.^3^^,^^4^ More recent research efforts have focused on the molecular mechanisms regulating satellite cell function in health and during a number of extreme experimental conditions (chemically-induced injury, crush, synergist ablation, genetic disease models).^4^^,^^5^ While there is no disputing the importance and relevance of these studies to understanding satellite cell biology, the translatability of these findings to more moderate activities/stressors which may be encountered more routinely in everyday life remain less clear.

Within this investigative framework entered a new mouse model, the Pax7CreER; R26DTA/+ (Pax7-DTA) mouse. This mouse model allows for the specific and inducible depletion of satellite cells upon tamoxifen treatment. Using this approach, Peterson and colleagues have revisited a number of the earlier studies investigating the importance of satellite cells to many facets of skeletal muscle health. Collectively their studies have demonstrated that satellite cells are not requisite for hypertrophy in the short term, but they are essential for sustained muscle growth in the long term.^^6–8^^ While these more recent investigations have greatly increased our understanding of the intrinsic and extrinsic mechanisms underlying the complexity of satellite cell behavior, and the particular stressors where satellite cells are essential for muscle health, an understanding of the relationship between the satellite cell and the myonuclei remained a fundamental gap in our current understanding. It is within this context where the present study from Englund et al. provides insight.^1^ By using their satellite cell-depleted mouse model and the physiological stress of progressive weighted running (which they termed PoWeR) they were able to investigate the contributions of satellite cells to an exercise-induced, hypertrophic stimulus. What the authors found was, that while satellite cell depleted soleus and plantaris muscles can adapt to a considerable degree in response to exercise, ultimately, muscle growth, strength, capillarization, and collagen remodeling were blunted when compared to satellite cell replete muscle. Transcriptional profiling of the skeletal muscles at multiple time points over the course of adaptation revealed several exercise-induced pathways were dysregulated in the absence of satellite cells, potentially contributing to the attenuated adaptation. This is the first study to examine how satellite cell content influences the myonuclear transcriptome in response to exercise, enabling the identification of pathways transcriptionally regulated by myonuclei (eg, ribosome biogenesis and metabolic adaptation) vs processes regulated by other cell types in the muscle compartment that promote adaptation to exercise. This integrative approach not only allowed for a more complete understanding of how muscles respond to exercise in the presence or absence of satellite cells, but also how the transcriptome of the muscle can be impacted by the presence or absence of satellite cells.

In many ways, this work is an elegant follow-up to the work of Murach et al.^9^ who demonstrated that satellite cells can communicate with surrounding muscles, capillaries, and fibroblasts via extracellular vesicles; in essence, satellite cells are more than just myonuclei in waiting, capable of affecting muscle health in a fusion-independent fashion.

Taken together, the original article by Englund et al. within this issue of *Function*^1^ provides us one of the few more complete pictures of the satellite cell’s influence on skeletal muscle in response to exercise training. Many questions remain, or are raised by the present study, such as: (1) Not all muscles respond similarly within this study. Is this a fiber type-specific effect or an intrinsic effect of the different stress that each of the muscles are exposed to? (2) Why did the remaining satellite cells (not cleared by tamoxifen) remain unresponsive to the PoWeR stimulus? Are they a unique population of Pax7-positive cells? Are they satellite cells that are insensitive to this type of stimulus? While this study was certainly a big leap forward for our appreciation of satellite cell biology, these unanswered questions (and others) provide fertile ground for future research.
